# Assessment of the Antioxidant and Reactive Oxygen Species Scavenging Activity of Methanolic Extract of *Caesalpinia crista* Leaf

**DOI:** 10.1093/ecam/nep072

**Published:** 2011-06-18

**Authors:** Sourav Mandal, Bibhabasu Hazra, Rhitajit Sarkar, Santanu Biswas, Nripendranath Mandal

**Affiliations:** Division of Molecular Medicine, Bose Institute, P-1/12 CIT Scheme VIIM, Kolkata-700054, India

## Abstract

“Oxidative stress” is initiated by reactive oxygen species (ROS), which are responsible for majority of the diseases. However, antioxidants with ROS scavenging ability may have great relevance in the prevention of oxidative stress. The present study was undertaken, using a 70% methanolic extract of *Caesalpinia crista* leaves, to examine different *in vitro* tests in diversified fields including total antioxidant activity, scavenging activities for various ROS, iron chelating activity and phenolic and flavonoid contents. Total antioxidant activity was evaluated as trolox equivalent antioxidant capacity value of 0.546 ± 0.014. The extract was investigated for different ROS scavenging activities and IC_50_ values were found to be 0.44 ± 0.1 mg/ml, 24.9 ± 0.98 **μ**g/ml, 33.72 ± 0.85 **μ**g/ml, 61.13 ± 3.24 **μ**g/mL and 170.51 ± 4.68 **μ**g/mL for hydroxyl, superoxide, nitric oxide, singlet oxygen and hypochlorous acid, respectively; however, no significant results were obtained in scavenging of hydrogen peroxide and peroxynitrite anion. The extract was found to be a potent iron chelator with IC_50_ = 279.85 ± 4.72 **μ**g/mL. The plant extract (100 mg) yielded 50.23 ± 0.003 mg/mL gallic acid equivalent phenolic content and 106.83 ± 0.0003 mg/mL quercetin equivalent flavonoid content. In the *in vivo* experiments, the extract treatment showed significant increase in the level of superoxide dismutase, catalase, glutathione-*S*-transferase and reduced glutathione. In a word, it may be concluded that 70% methanol extract of *C. crista* leaves acts as an antioxidant and ROS scavenger; which may be due to the presence of phenolic and flavonoid compounds.

## 1. Introduction

Reactive oxygen species (ROS), generated in cells, are fundamental in modulating various physiological functions and represent an essential part of aerobic life and metabolism [1]. Sometimes, excessive generation of these radicals disrupts the antioxidant defense system of the body which may lead to “oxidative stress”. This situation brings about a variety of disorders including coronary heart disease, neurodegenerative disorders, diabetes, arthritis, inflammation, lung damage and cancer [2, 3]. Since, antioxidants are capable of preventing oxidative damage, the wide use of natural antioxidants as a replacement of conventional synthetic antioxidants in food and food supplements has been employed owing to the fact that natural products are considered to be promising and safe source.

*Caesalpinia crista* Linn. (syn. *C. bonducella* [L.] Roxb.) (family—Fabaceae) is a popular medicinal plant widely distributed throughout the tropical and subtropical regions of Southeast Asia. It is found throughout the hot and humid part of India, Myanmar and Sri Lanka. In India, it is known as *Katikaranja* and is distributed in West Bengal, Kerala and Maharashtra. It is a large straggling and very thorny shrub. Medicinally, most widely used part is seed kernel which is reported as a rich source of cassane- and norcassane-type diterpenoids [4]. Some new diterpenoids are also isolated from stems and root of this plant [5]. The plant is locally known as *Ka*-*Lain* in Myanmar and its seeds are used as anthelmintic, antipyretic, anti-inflammatory and antimalarial agent. In Indonesia, it is known as *Bagor* and decoction of root have been used as a tonic for the treatment of rheumatism. Traditionally, in Ayurveda, this plant was used for the treatment of gynecological disorders, skin diseases, constipation, piles and ulcers [6]. It is also reported to have antidiuretic, antibacterial, antianaphylactic, antidiarrhoeal, antiameobic and antiviral [7, 8] properties. It also possesses antidiabetic and hypoglycaemic activity [9].

It has been reported that the methanol extract of *C. crista* seed and seed kernel possess antifeedant [10] and anthelmintic [11] property. There is still no report on the antioxidant property of this plant leaf. The present study is aimed to evaluate the antioxidant potential and ROS scavenging activity from the 70% methanol extract of *C. crista* leaf.

## 2. Methods

### 2.1. Chemicals

2,2′-Azinobis-(3-ethylbenzothiazoline-6-sulfonic acid) (ABTS) was obtained from Roche Diagnostics, Mannheim, Germany. 6-Hydroxy-2,5,7,8-tetramethylchroman-2-carboxylic acid (Trolox) was obtained from Fluka, Buchs, Switzerland. 2-Deoxy-2-ribose, trichloroacetic acid (TCA), mannitol, nitro blue tetrazolium (NBT), reduced nicotinamide adenine dinucleotide phosphate (NADH), phenazine methosulfate (PMS), sodium nitroprusside (SNP), sulfanilamide, naphthylethylenediamine dihydrochloride (NED), l-histidine, lipoic acid, sodium pyruvate, quercetin, glutathione reduced, 5,5′-dithiobis-2-nitrobenzoic acid (DTNB), 1-chloro-2,4-dinitrobenzene (CDNB) and ferrozine were obtained from Sisco Research Laboratories Pvt. Ltd, Mumbai, India. Potassium hexacyanoferrate, Folin-Ciocalteu reagent, butylated hydroxy toluene (BHT), sodium hypochlorite (NaOCl), aluminum chloride (AlCl_3_), xylenol orange and *N*,*N*-dimethyl-4-nitrosoaniline were obtained from Merck, Mumbai, India. Gallic acid and curcumin were obtained from MP Biomedicals, France. HEPES buffer and catalase were obtained from HiMedia Laboratories Pvt. Ltd, Mumbai, India. Evans Blue was purchased from BDH, England. Diethylene-triamine-pentaacetic acid (DTPA) was obtained from Spectrochem Pvt. Ltd, Mumbai, India. Thiobarbituric acid (TBA) was obtained from Loba Chemie, Mumbai, India.

### 2.2. Plant Material

The leaves of *C. crista* were collected from the Bankura district of West Bengal, India. It was identified and authenticated through the Central Research Institute (Ayurveda), Kolkata, India and a voucher specimen (CRHS 121/08) was submitted there.

### 2.3. Animals

Male Swiss albino mice (20 ± 2 g) were purchased from Chittaranjan National Cancer Institute (CNCI), Kolkata, India and were maintained under a constant 12-h dark/light cycle at an environmental temperature of 22 ± 2°C. The animals were provided with normal laboratory pellet diet and water *ad libitum*. All experiments were performed after obtaining approval from the institutional animal ethics committee.

### 2.4. Sample Preparation

The powder (100 g) of the dried leaves of *C. crista* was stirred using a magnetic stirrer with 500-mL mixture of methanol : water (7 : 3) for 15 h; then the mixture was centrifuged at 2850 g and the supernatant decanted. The process was repeated again with the precipitated pellet. The supernatants were collected, concentrated in a rotary evaporator and lyophilized. The dried extract was stored at −20°C until use.

### 2.5. In Vitro Antioxidant Assay

#### 2.5.1. Trolox Equivalent Antioxidant Capacity (TEAC)

Antioxidant activity of the extract was assayed depending on the ability of the sample to scavenge ABTS^•+^ radical cation compared to trolox standard [12]. The ABTS^•+^ radical cation was pregenerated by mixing 7-mM ABTS stock solution with 2.45-mM potassium persulfate (final concentration) and incubated for 12–16 h in dark at room temperature until the reaction was completed and absorbance was stable. The absorbance of ABTS^•+^ was equilibrated to 0.70 (±0.02) by diluting with water at room temperature. Then 10 *μ*L (0.05–10 mg/mL) sample solution was mixed with 1-mL ABTS^•+^ solution and after 6 min, absorbance was measured at 734 nm. All experiments were repeated six times. The percentage inhibition of absorbance was calculated and plotted as a function of the concentration of standard and sample to determine the TEAC. TEAC was calculated from dividing the gradient of the plot for the sample by the gradient of the plot for trolox.

#### 2.5.2. Hydroxyl Radical Scavenging Activity

This assay was performed by a standard method [13] with a slight modification. Hydroxyl radical was generated by the Fe^3+^-ascorbate-EDTA-H_2_O_2_ system (Fenton reaction). The assay is based on the quantification of the 2-deoxyribose degradation product, by its condensation with TBA. The reaction mixture contained, in a final volume of 1 mL, 2-deoxy-2-ribose (2.8 mM); KH_2_PO4–KOH buffer (20 mM, pH 7.4); FeCl_3_ (100 *μ*M); EDTA (100 *μ*M); H_2_O_2_ (1.0 mM); ascorbic acid (100 *μ*M) and various concentrations (0–1 mg/mL) of test sample or reference compound. After incubation at 37°C for 1 h, 1 mL 2.8% TCA was added to 0.5 mL of the reaction mixture. Then 1-mL 1% aqueous TBA was added to the solution and incubated at 90°C for 15 min to develop the color. After cooling, the absorbance was measured at 532 nm against the blank solution. All tests were performed six times. Mannitol, a classical ^•^OH scavenger, was used as a positive control. Percentage inhibition was evaluated by comparing the results of the test and blank solution.

#### 2.5.3. Scavenging of Superoxide Radicals

This activity was done based on the reduction of NBT according to a previously reported method [14]. The non-enzymatic phenazine methosulfate-nicotinamide adenine dinucleotide (PMS/NADH) system generates superoxide radicals that reduce nitro blue tetrazolium (NBT) into a purple-colored formazan. The 1-mL reaction mixture contained phosphate buffer (20 mM, pH 7.4), NADH (73 *μ*M), NBT (50 *μ*M), PMS (15 *μ*M) and various concentrations (0–50 *μ*g/mL) of sample solution. After incubation for 5 min at ambient temperature, the absorbance was taken at 562 nm against an appropriate blank solution. All tests were performed six times. Quercetin was used as positive control.

#### 2.5.4. Assay of Nitric Oxide Radical Scavenging

At physiological pH, nitric oxide generated from aqueous sodium nitroprusside (SNP) solution interacts with oxygen to produce nitrite ions which were measured by Griess Illosvoy reaction [15]. The 3-mL reaction mixture contained 10-mM SNP, phosphate buffered saline (pH 7.4) and various doses (0–120 *μ*g/mL) of test solution. After incubation for 150 min at 25°C, 1-mL sulfanilamide (0.33% in 20% glacial acetic acid) was added to 0.5 mL of the incubated solution and allowed to stand for 5 min. Then 1 mL of napthylethylenediamine dihydrochloride (NED) (0.1% w/v) was added and the mixture was incubated for 30 min at 25°C. The pink chromophore formed during diazotization of nitrite ions with sulphanilamide and subsequent coupling with NED was measured spectrophotometrically at 540 nm against blank sample. All tests were performed six times. Curcumin was used as a standard.

#### 2.5.5. Scavenging Activity of Hydrogen Peroxide

This activity was determined according to a method [16] with minor changes. Aliquot of 50-mM H_2_O_2_ and various concentrations (0–2.0 mg/mL) of samples were mixed (1 : 1 v/v) and incubated for 30 min at room temperature. After incubation, 90 *μ*l of the H_2_O_2_-sample solution was mixed with 10 *μ*l HPLC-grade methanol and 0.9-mL FOX reagent was added (previously prepared by mixing 9 volumes of 4.4-mM BHT in HPLC-grade methanol with 1 volume of 1-mM xylenol orange and 2.56-mM ammonium ferrous sulfate in 0.25-M H_2_SO_4_). The reaction mixture was then vortexed and incubated at room temperature for 30 min. The absorbance of ferric-xylenol orange complex was measured at 560 nm. All tests were carried out six times and sodium pyruvate was used as the reference compound.

#### 2.5.6. Determination of the Effects on Peroxynitrite

Peroxynitrite (ONOO^−^) was synthsized following a method by Beckman et al. [17]. An acidic solution (0.6-M HCl) of 5-mL H_2_O_2_ (0.7M) was mixed with 5-mL 0.6 M KNO_2_ on an ice bath for 1 s and 5-mL ice-cold 1.2-M NaOH was added to the reaction mixture. Excess H_2_O_2_ was removed by treatment with granular MnO_2_ prewashed with 1.2-M NaOH and the reaction mixture was left overnight at −20°C. Peroxynitrite solution was collected from the top of the frozen mixture and the concentration was measured spectrophotometrically at 302 nm (*ε* = 1670/M cm).

The Evans Blue bleaching assay [18] was used to measure the peroxynitrite scavenging activity, with slight modification. The reaction mixture contained 50-mM phosphate buffer (pH 7.4), 0.1-mM DTPA, 90-mM NaCl, 5-mM KCl, 12.5 *μ*M Evans Blue, various doses of plant extract (0–200 *μ*g/mL) and 1-mM peroxynitrite in a final volume of 1 mL. After incubation at 25°C for 30 min the absorbance was measured at 611 nm. The percentage of scavenging of ONOO^−^ was calculated by comparing the results of the test and blank sample. All tests were performed six times. Gallic acid was used as reference compound.

#### 2.5.7. Reaction with Singlet Oxygen

The production of singlet oxygen (^1^O_2_) was determined by monitoring *N*,*N*-dimethyl-4-nitrosoaniline (RNO) bleaching, using a previously reported spectrophotometric method [19]. Singlet oxygen was generated by a reaction between NaOCl and H_2_O_2_ and the bleaching of RNO was read at 440 nm. The reaction mixture contained 45-mM phosphate buffer (pH 7.1), 50-mM NaOCl, 50-mM H_2_O_2_, 50-mM l-histidine, 10 *μ*M RNO and various concentrations (0–100 *μ*g/mL) of sample in a final volume 2 mL. It was incubated at 30°C for 40 min and decrease in the absorbance of RNO was measured at 440 nm. The scavenging activity of sample was compared with that of lipoic acid, used as a reference compound. All tests were performed six times.

#### 2.5.8. Reaction with Hypochlorous Acid

Hypochlorous acid (HOCl) was prepared just before the experiment by adjusting the pH of a 10% (v/v) solution of NaOCl to 6.2 with 0.6 M H_2_SO_4_ and the concentration of HOCl was determined by using the absorbance at 235 nm and the molar extinction coefficient of 100/M cm.

The assay was carried out as described by Aruoma and Halliwell [20] with minor changes. The scavenging activity was evaluated by measuring the decrease in absorbance of catalase at 404 nm. The reaction mixture contained, in a final volume of 1 mL, 50-mM phosphate buffer (pH 6.8), catalase (7.2 *μ*M), HOCI (8.4 mM) and increasing concentrations (0–200 *μ*g/mL) of plant extract. The assay mixture was incubated at 25°C for 20 min and the absorbance was taken against an appropriate blank. All tests were performed six times. Ascorbic acid was used as the reference compound.

#### 2.5.9. Fe^2+^ Chelation

The chelating activity of the extracts for ferrous ion was evaluated by a standard method [21] with minor changes. The reaction was carried out in HEPES buffer (20 mM, pH 7.2). Briefly, various concentrations of plant extracts (0–300 *μ*g/mL) were added to 12.5 *μ*M ferrous sulfate solution and the reaction was initiated by the addition of ferrozine (75 *μ*M). The mixture was shaken vigorously and incubated for 20 min at room temperature, and the absorbance was measured at 562 nm. All tests were performed for six times. EDTA was used as a positive control.

#### 2.5.10. Measurement of Reducing Power

The Fe^3+^ reducing power of the extract was determined by the method of Oyaizu [22] with a slight modification. Different concentrations (0–1.0 mg/mL) of extract (0.5 mL) were mixed with 0.5-mL  sphate buffer (pH 6.6) and 0.5-mL 0.1% potassium hexacyanoferrate, followed by incubation at 50°C in water bath for 20 min. After incubation, 0.5-mL 10% TCA was added to terminate the reaction. The upper portion of the solution (1 mL) was mixed with 1 mL of distilled water and 0.1-mL 0.01% FeCl_3_ solution was added. The reaction mixture was left for 10 min at room temperature and the absorbance was measured at 700 nm against appropriate blank solution. All tests were performed six times. A higher absorbance of the reaction mixture indicated greater reducing power. Ascorbic acid was used as a positive control.

#### 2.5.11. Determination of Total Phenolic Content

Total phenolic content was determined using Folin-Ciocalteu (FC) reagent according to the method of Singleton and Rossi [23] with a slight modification. Briefly, 0.1 mL of extract was mixed with 0.75 mL of FC reagent (previously diluted 1000-fold with distilled water) and incubated for 5 min at 22°C; then 0.06% Na_2_CO_3_ solution was added to the mixture. After incubation at 22°C for 90 min, the absorbance was measured at 725 nm. The phenolic content was evaluated from a gallic acid standard curve.

#### 2.5.12. Determination of Total Flavonoids

Total flavonoid content was decided according to a known method [24] using quercetin as a standard. The plant extract of 0.1 mL was added to 0.3-mL distilled water followed by 0.03-mL 5% NaNO_2_. After 5 min at 25°C, 0.03-mL 10% AlCl_3_ was added. After another 5 min, the reaction mixture was treated with 0.2-mL 1-mM NaOH. Finally the reaction mixture was diluted to volume (1 mL) with water. Then the absorbance was measured at 510 nm. The flavonoid content was calculated from a quercetin standard curve.

### 2.6. In Vivo Antioxidant Assay

#### 2.6.1. Experimental Design

Animals were divided into four groups containing six animals in each group. Group I animals served as control and received normal saline only. Groups II, III and IV received the plant extract at a dose of 10, 50 and 100 mg/kg body weight, respectively. The treatments were carried out orally for 7 days and on the 8th day all the animals were sacrificed by cervical dislocation. The liver was rapidly removed and after washing with ice-cold saline it was homogenized in 10 volume of 0.1-M phosphate buffer (pH 7.4) containing 5-mM EDTA and 0.15-M NaCl, and centrifuged at 8000 g for 30 min at 4°C. The supernatant was collected and used for the assay of enzyme activities. Protein concentration was estimated according to Lowry method [25] using BSA as standard.

#### 2.6.2. Assay of Antioxidant Enzymes

Superoxide dismutase (SOD) was assayed by measuring the inhibition of the formation of blue colored formazan at 560 nm according to the technique of Kakkar et al. [26]. Catalase (CAT) activity was measured by following the decrease in H_2_O_2_ concentration spectrophotometrically over time at 240 nm according to a previously described method [27]. Glutathione-*S*-transferase (GST) was determined by the method of Hobig et al. [28] based on the formation of GSH-CDNB conjugate and increase in the absorbance at 340 nm. Reduced glutathione (GSH) level was measured spectrophotometrically at 412 nm by the method of Ellman [29].

### 2.7. Statistical Analysis

All data are reported as the mean ± SD of six measurements. Statistical analysis was performed using KyPlot version 2.0 beta 15 (32 bit). The IC_50_ values were calculated using the formula *Y* = 100 × A1/(*X* + A1) where A1 = IC_50_, *Y* = response (*Y* = 100% when *X* = 0), *X* = inhibitory concentration. The IC_50_ values were compared by paired *t*-test. The results with a value of *P* < .05 were considered significant.

## 3. Results

### 3.1. In Vitro Antioxidant Assay

#### 3.1.1. Total Antioxidant Activity

**Figure 1:**
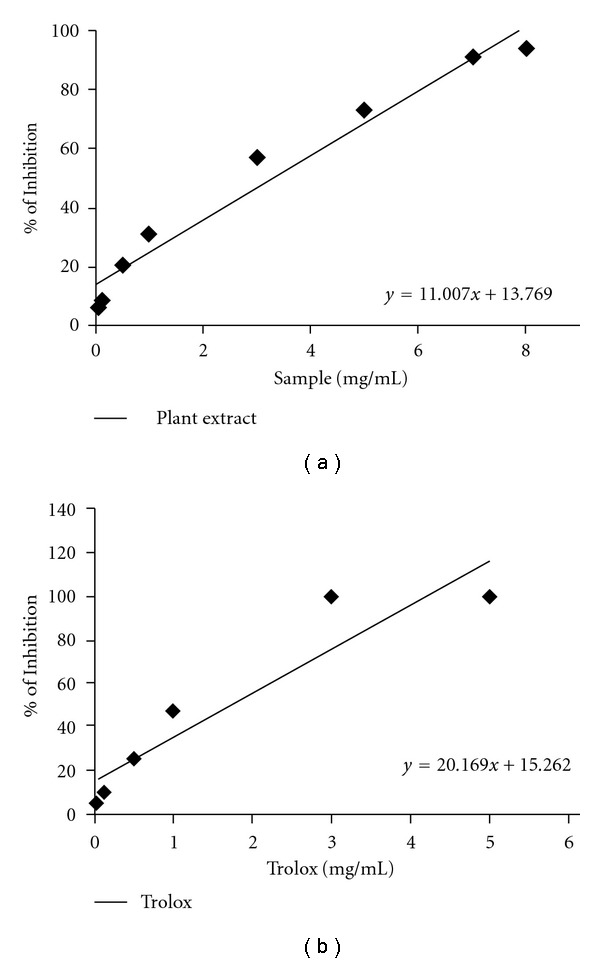
Total antioxidant activity. Effects of (a) *C. crista* leaf extract and (b) reference compound trolox on ABTS radical cation decolorization assay. The percentage of inhibition is plotted against concentration of sample. The value represented as mean ± SD (*n* = 6).

The total antioxidant activity of *C. crista* extract was calculated from the decolorization of the ABTS^•+^, which was measured spectrophotometrically at 734 nm. The percentage inhibition of absorbance was calculated and plotted as a function of concentration of the extract and of standard trolox, as shown in Figures 1(a) and 1(b), respectively. The TEAC value of the extract was found to be 0.546 ± 0.014.

#### 3.1.2. Inhibition of ^•^OH-Induced Deoxyribose Degradation

**Figure 2:**
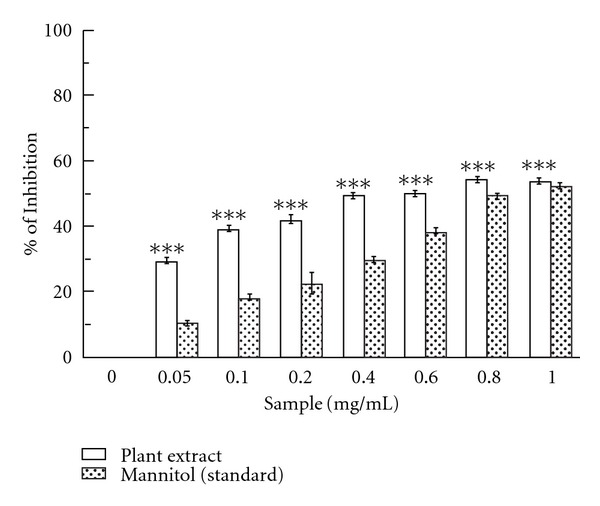
Inhibition of deoxyribose degradation. Hydroxyl radical scavenging activities of the *C. crista* leaf extract and the reference compound mannitol. The data represent the percentage of inhibition of deoxyribose degradation. ***P < .001 versus 0 mg/mL.

The ability of the extract and standard mannitol to inhibit hydroxyl radical mediated deoxyribose degradation in Fe^3+^-EDTA-ascorbic acid and H_2_O_2_ reaction mixture was shown in this assay. The results have been shown in Figure 2. The IC_50_ value (Table 1) of extract and standard in hydroxyl radical scavenging assay was 0.44 ± 0.1 mg/mL and 0.85 ± 0.02 mg/mL, respectively. At the concentration of 1 mg/mL, the percentage inhibition was 53.75 and 52.05% for *C. crista* and mannitol, respectively.

#### 3.1.3. Potent Superoxide Scavenger *C. crista*

**Figure 3:**
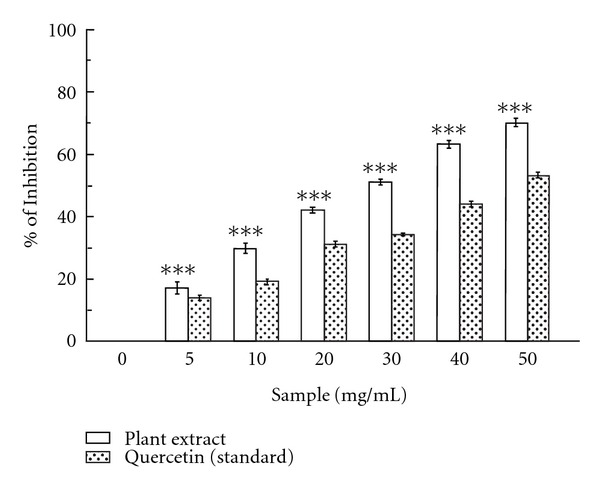
Superoxide radical scavenging activity. Scavenging effects of *C. crista* leaf extract and the standard quercetin on superoxide radical. The data represent the percentage of superoxide radical inhibition. ***P < .001 versus 0 *μ*g/mL.

PMS-NADH coupling yields superoxide radicals that can be measured by their ability to reduce NBT. The abilities of the plant extract and the reference compound quercetin to quench superoxide radicals from reaction mixture is reflected in the decrease of the absorbance at 560 nm. As shown in Figure 3, the IC_50_ values (Table 1) of plant extract and quercetin on superoxide scavenging activity were found to be 24.9 ± 0.98 *μ*g/mL and 47.38 ± 0.68 *μ*g/mL, respectively. At 50 *μ*g/mL, the percentage inhibition of the plant extract was 70.23%, whereas that of quercetin was 53.4%.

#### 3.1.4. Nitric Oxide Radical Scavenging Ability

**Figure 4:**
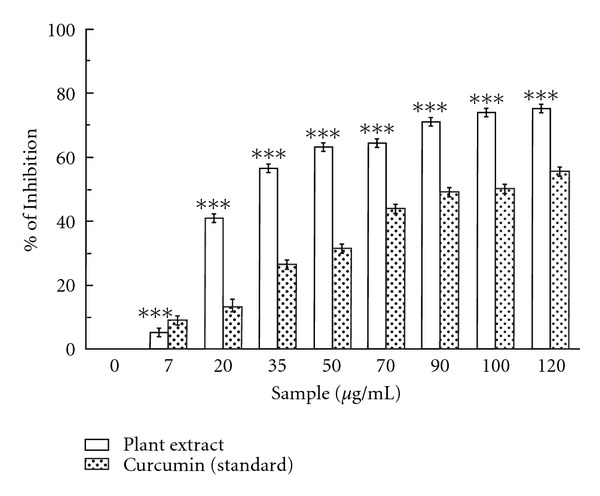
Nitric oxide radical scavenging activity. Nitric oxide radical scavenging activities of *C. crista* leaf extract and standard curcumin. The data represent the precentage of nitric oxide inhibition. ***P < 0.001 versus 0 *μ*g/mL.

*Caesalpinia crista* extract also superbly inhibits nitric oxide in dose dependent manner (Figure 4) with the IC_50_ (Table 1) value being 33.72 ± 0.85 *μ*g/mL. Curcumin was used as a reference compound and 97.12 ± 3.09 *μ*g/mL curcumin was needed for 50% inhibition. The IC_50_ value of the extract was lesser than that of the standard. At 120 *μ*g/mL, the percentage inhibition of the plant extract was 75.26%, whereas that of curcumin was 55.68%.

#### 3.1.5. Hydrogen Peroxide Scavenging

The scavenging activity for hydrogen peroxide of *C. crista* was not at all worth acknowledgeable in the assay performed by the FOX reagent method. The IC_50_ value for the plant extract was found to be indeterminable, whereas that of the standard sodium pyruvate was 3.24 ± 0.30 mg/mL. The data are not shown.

#### 3.1.6. Peroxynitrite Scavenging Activity

*Caesalpinia crista* extract did not show any noteworthy result in scavenging peroxynitrite radical, in comparison to the standard gallic acid (IC_50_ = 876.24 ± 56.96 *μ*g/mL). So, the results and the figures are not provided.

#### 3.1.7. Inhibition of RNO Bleaching

**Figure 5:**
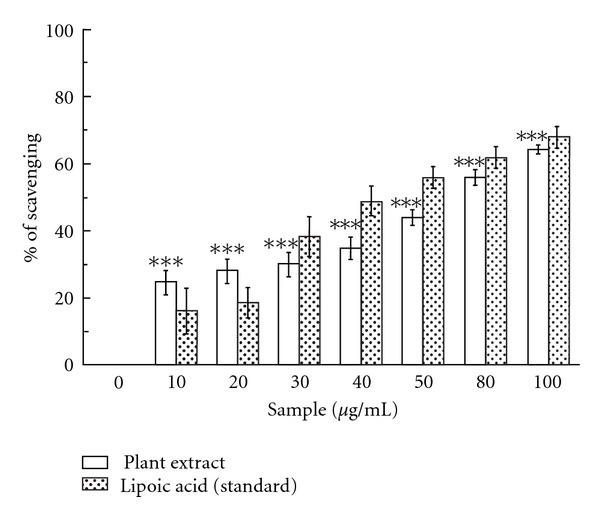
Inhibition of RNO bleaching. Effects of *C. crista* leaf extract and standard lipoic acid on the scavenging of singlet oxygen. ***P < .001 versus 0 *μ*g/mL.

*Caesalpinia crista* extract was an effective scavenger of singlet oxygen (Figure 5) and this activity was comparable to that of lipoic acid. The IC_50_ value (Table 1) of the test sample was found to be 61.13 ± 3.24 *μ*g/mL, whereas the IC_50_ value of lipoic acid was found to be 49.48 ± 8.56 *μ*g/mL. At 100 *μ*g/mL, the percentage of scavenging of the plant extract was 64.18% whereas that of lipoic acid was 67.7%.

#### 3.1.8. Hypochlorous Acid Scavenging Activity

**Figure 6:**
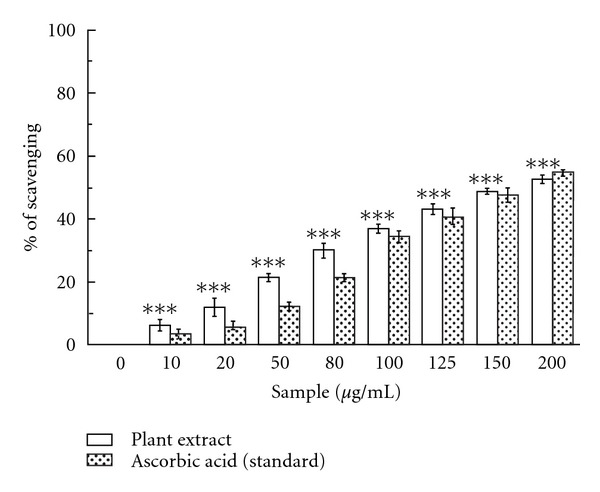
Hypochlorous acid scavenging activity. Hypochlorous acid scavenging activities of *C. crista* leaf extract and standard ascorbic acid. ***P < .001 versus 0 *μ*g/mL.

Figure 6 shows the dose-dependent hypochlorous acid scavenging activity of *C. crista* extract compared to that of ascorbic acid. The obtained results indicate that the extract scavenged hypochlorous acid more efficiently (IC_50_ = 170.51 ± 4.68 *μ*g/mL) than ascorbic acid (IC_50_ = 198.42 ± 10.71 *μ*g/mL) (Table 1). At 200 *μ*g/mL, the percentage of scavenging of the plant extract was 52.64%, whereas that of ascorbic acid was 54.91%.

#### 3.1.9. Inhibition of Fe^2+^-Ferrozine Formation

**Figure 7:**
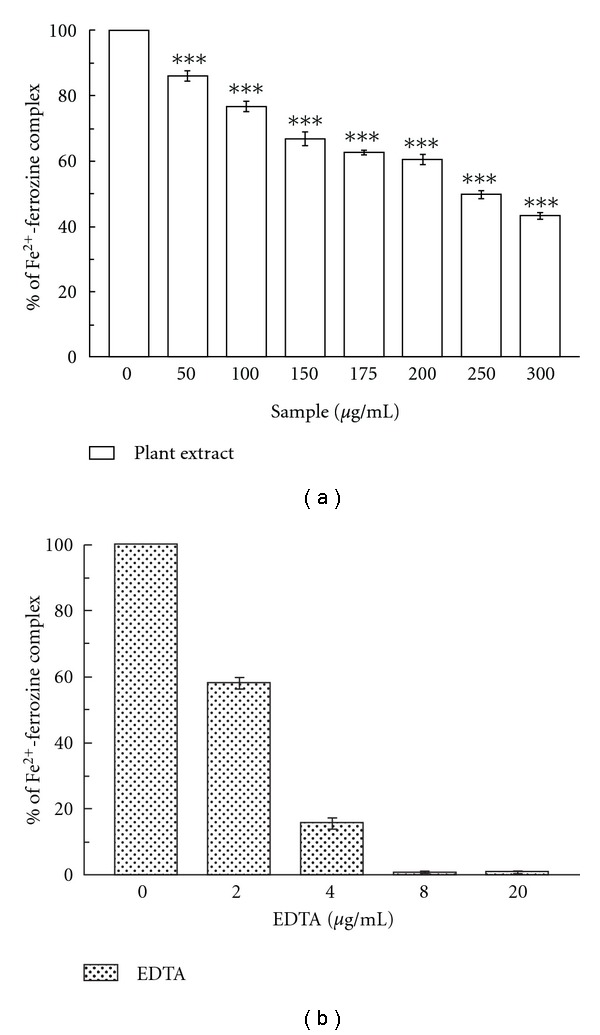
Inhibition of Fe^2+^–ferrozine formation. Effects of (a) *C. crista* leaf extract and (b) standard EDTA on ferrozine-Fe^2+^ complex formation. The data expressed as percentage inhibition of chromogen formation. ***P < .001 versus 0 *μ*g/mL.

Ferrozine makes a violet colored complex with Fe^2+^ ion. The complex formation is interrupted in presence of chelating agent and as a result the violet color of the complex is decreased. The results (Figures 7(a) and 7(b)) demonstrated that the formation of ferrozine-Fe^2+^ complex is inhibited in the presence of test and reference compound. The IC_50_ values (Table 1) of the plant extract and EDTA were 279.85 ± 4.72 *μ*g/mL and 1.27 ± 0.05 *μ*g/mL, respectively. At 300 *μ*g/mL, the percentage of inhibition of complex formation by the plant extract was 43.33%, whereas at 45 *μ*g/mL that of EDTA was 99.5%.

#### 3.1.10. Total Reduction Capability

**Figure 8:**
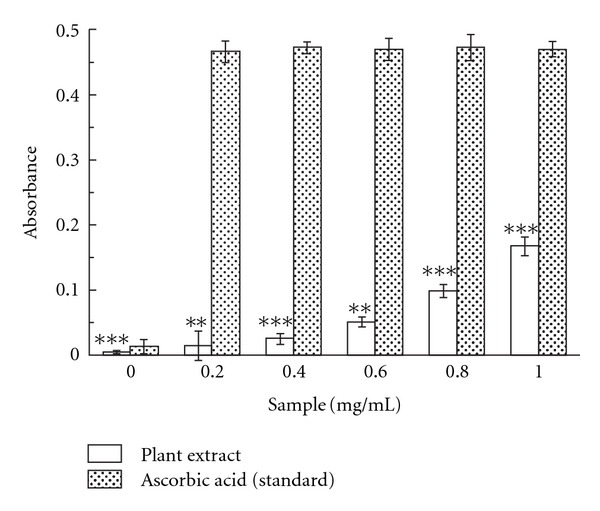
Total reduction capability. The reductive ability of *C. crista* leaf extract and standard ascorbic acid. The absorbance (A_700_) was plotted against concentration of sample. **P < .01 and ***P < .001 versus 0 mg/mL.

As illustrated in Figure 8, from Fe^3+^ to Fe^2+^ transformation in the presence of *C. crista* extract and reference ascorbic acid was performed to measure the reductive capability. Although the activity of ascorbic acid was better than the sample with absorbance values of 0.47 and 0.167 for the reference and sample, respectively, still the sample showed somewhat moderate reducing capability.

#### 3.1.11. Amount of Total Phenolic Content

Phenolic compounds may be directly related to the antioxidative action. The total phenolic content was 50.23 ± 0.003 mg/mL gallic acid equivalent per 100-mg plant extract.

#### 3.1.12. Amount of Total Flavonoids

Total flavonoid content of the 70% methanolic extract of *C. crista* was 106.83 ± 0.0003 mg/mL quercetin per 100-mg plant extract.

### 3.2. In Vivo Antioxidant Assay

#### 3.2.1. Effect on Enzyme Activity

The oral administration of plant extract to normal mice for 7 days significantly enhanced the activity of SOD in a dose dependent manner. The results are significant (*P* < .001 at the dose of 50 and 100 mg/kg body weight and *P* < .01 at the dose of 10 mg/kg body weight) when compared with control (Table 2). The treatment induced catalase activity significantly (*P* < .05) at the dose of 50 and 100 mg/kg body weight (Table 2). The level of GST was also increased significantly (Table 2) at the dose of 50 and 100 mg/kg body weight (*P* < .05 and *P* < .001, resp., compared with control). The dose-dependent augment of GSH content (Table 2) was also found to be significant (*P* < .05 at 10 mg/kg body weight and *P* < .001 at 50 and 100 mg/kg body weight).

## 4. Discussion

ROS are responsible for the damage of cellular bio-molecules such as proteins, enzymes, nucleic acids, lipids and carbohydrates and may adversely affect immune functions [30]. Antioxidants interrupt the production of ROS and also play a key role to inactivate them. Although, all human cells protect themselves against oxidative damage by some antioxidant mechanism, these sometimes are not sufficient to prevent the ROS damage totally. Different kind of plant materials have already been reported as natural antioxidants [31, 32]. Recently, we have also shown the antioxidant and ROS scavenging activity of *Spondias pinnata* bark [33].

In Ayurveda, the seeds, root-bark and the leaves of *C. crista* are used for medicinal purpose. Externally, the paste of leaves gives great relief from the pain and edema. Internally, it is the best panacea of abdominal pain, diarrhea, dysentery and colitis. The present results of the study demonstrate that 70% methanolic extract of *C. crista* has potent antioxidant activity and ROS scavenging activity as well as iron chelating property.

The reaction between ABTS and potassium persulfate results in the production of a blue colored chromophore, ABTS^•+^. After addition of the plant extract this pre-formed radical cation was converted to ABTS in a dose dependant manner. The result is compared with trolox and the TEAC value demonstrates the extract as a potent antioxidant.

Hydroxyl radical is one of the ROS formed in biological systems, causing DNA strand breakage, which brings about carcinogenesis, mutagenesis and cytotoxicity [34]. Fe^3+^-EDTA premixture is incubated with ascorbic acid and H_2_O_2_ at pH 7.4. Thus, hydroxyl radicals are formed, which cause 2-deoxy-2-ribose damage and generate malondialdehyde (MDA) like product. This compound forms a pink chromogen upon heating with TBA at low pH [35]. Addition of the *C. crista* extract to the reaction mixture removes hydroxyl radicals and prevents further damage. The observed IC_50_ value indicates that the plant extract is a better hydroxyl radical scavenger than standard mannitol.

Superoxide anion is also implicated as harmful ROS. It has detrimental effect on the cellular components in a biological system [36]. It indirectly initiates lipid oxidation by generating singlet oxygen. The dose dependent increase in the scavenging activity of the plant extract and the standard quercetin for superoxide radical (Figure 3) suggest that the former is more potent scavenger than the latter.

The production of nitric oxide radical at a sustained level results in direct tissue toxicity and contribute to the vascular collapse associated with septic shock, whereas chronic expression of nitric oxide radical is associated with various carcinomas and inflammatory conditions including juvenile diabetes, multiple sclerosis, arthritis and ulcerative colitis [37]. The reaction of NO with superoxide radical generates highly reactive peroxynitrite anion (ONOO^−^) which is highly toxic for living cell [38]. The nitric oxide generated from sodium nitroprusside reacts with oxygen to form nitrite. The extract directly competes with oxygen to react with nitric oxide, thus inhibiting nitrite formation. The present study proved that the nitric oxide scavenging activity of the studied extract is better than the standard curcumin.

Another ROS, singlet oxygen which is a high energy form of oxygen, is generated in the skin upon UV-irradiation. Singlet oxygen induces hyperoxidation, oxygen cytotxicity and decreases the antioxidative activity [39]. The present study indicates that the *C. crista* extract has restrained scavenging activity for singlet oxygen but is not as efficient as the standard lipoic acid.

At the sites of inflammation, the oxidation of Cl^−^ ions by the neutrophil enzyme myeloperoxidase results in the production of another harmful ROS, hypochlorous acid [40]. HOCl has the ability to inactivate the antioxidant enzyme, catalase through break down of heme-prosthetic group. The inhibition of catalase inactivation in the presence of the extract signifies its HOCl scavenging activity and from the results obtained, it is anticipated that *C. crista* is the more efficient scavenger than standard ascorbic acid.

The two oxidation states of iron, Fe^2+^ and Fe^3+^ donate or accept electrons through redox reactions that are significant for biological reactions, but they also may be harmful to cells [41]. In excess, iron helps superoxide anion (O^•2−^) and hydrogen peroxide to convert into the extremely reactive hydroxyl radical (OH^•^) (Haber-Weiss reaction) that cause severe injury to membranes, proteins and DNA [2]. It decomposes lipid hydro-peroxides into peroxyl and alkoxyl radicals responsible for the chain reaction of lipid peroxidation [42]. The results from Figure 7 and Table 1 suggest that the decrease in the concentration dependent color formation with ferrozine in presence of extract indicating its iron chelating property. However, compared to standard EDTA, it shows less activity.

The reducing capacity of a compound may serve as a significant indicator of its potential antioxidant activity. As shown in Figure 8, it may be observed that the plant extract has some reducing capacity, thus justifying its antioxidant capacity.

It is also found that *C. crista* plant extract shows significant amount of flavonoid and phenolic content. Flavonoids show their antioxidative action through scavenging or chelating process [43]. Phenolic content is also very important plant constituent because of their scavenging ability due to their hydroxyl groups [44]. Both of these compounds have good antioxidant potential and their effects on human nutrition and health are significant.

It has been reported that body's antioxidant defense system consisting of the activity of SOD, CAT, GST and GSH [45]. SOD catalyzes the breakdown of endogenous cytotoxic superoxide radicals to H_2_O_2_ which is further degraded by CAT. Thus, they play a crucial role in maintaining the physiological levels of O_2_ and H_2_O_2_^*•*^ GSH, in conjunction with GST, has a basic role in cellular defense against deleterious free radicals and other oxidant species [46]. GST catalyzes the conjugation of thiol group of glutathione to electrophilic substrates, and thereby detoxifies endogenous compounds such as peroxidized lipids [47]. The present study supports the antioxidant potency of the plant extract as evidenced by the increased level of these antioxidant systems in extract treated mice.

## 5. Conclusions

**Figure 9:**
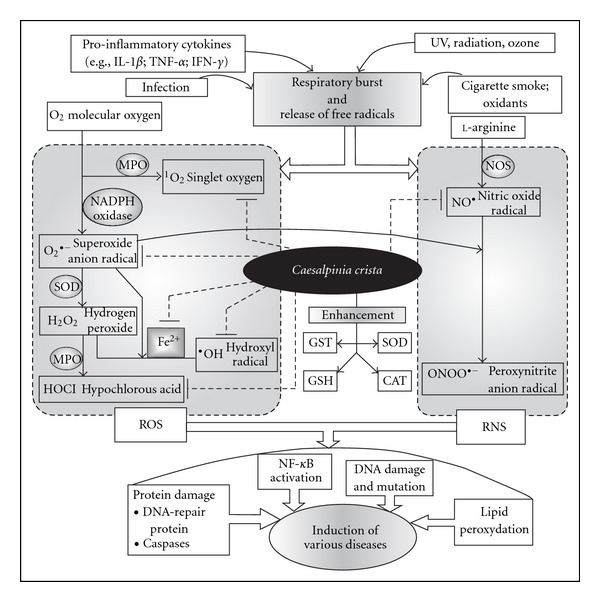
Role of *C. crista* in prevention of diseases caused by free radicals. Generation of ROS and reactive nitrogen species (RNS) is initiated by respiratory burst, which is set off by various physiological and environmental factors. The fabrication of an assortment of ROS and RNS from the molecular O_2_ and l-arginine, respectively, carried on by different enzymes like MPO (myloperoxidase), NADPH oxidase, SOD (superoxide dismutase) and NOS (nitric oxide synthase) leads to diverse cellular phenomena, namely, damage of DNA-repair proteins and caspases, lipid peroxydation, DNA damage followed by mutation and NF-*κ*B activation. All these phenomena give rise to wide range of diseases. *Caesalpinia crista* leaf extract inhibits the generations of the free radicals by scavenging both the mother and the daughter products and also by inducing the increase of SOD, CAT, GST and GSH, resulting the obstruction of various disease formation.

Conclusively, it can be avowed that the 70% methanolic extract of *C. crista* leaves, which contains high amount of flavonoid and phenolic contents, exhibits high antioxidant and ROS scavenging activity. It also has iron chelating property. Furthermore, evaluation of *in vivo* antioxidant activity of this extract has also provided interesting results that might be beneficial for the pharmacological use of this plant in clinical trials. In a word, these results signify that this plant extract is an important source of natural antioxidant, which might play a vital role in preventing the progress of various oxidative stresses, in course of enhancing the generation of typical antioxidant enzymes (Figure 9). Thus, these inspiring results provide the impetus to investigate the reason behind the antioxidative property.

## Figures and Tables

**Table 1:** Reactive oxygen species scavenging and iron chelating activity (IC_50_ values) of *C. crista* and reference compounds.

Activity	Extract/reference	IC_50_^a^
Hydroxyl radical (OH^•^) scavenging	*Caesalpinia crista*	0.44 ± 0.1 (6)
	Mannitol	0.85 ± 0.02 (6)***
Superoxide anion (O_2_^•−^) scavenging	*Caesalpinia crista*	24.9 ± 0.98 (6)
	Quercetin	47.38 ± 0.68 (6)***
Nitric oxide radical (NO) scavenging	*Caesalpinia crista*	33.72 ± 0.85 (6)
	Curcumin	97.12 ± 3.09 (6)***
Singlet oxygen (^1^O_2_) scavenging	*Caesalpinia crista*	61.13 ± 3.24 (6)
	Lipoic acid	49.48 ± 8.56 (6)**
Hypochlorous acid (HOCl) scavenging	*Caesalpinia crista*	170.51 ± 4.68 (6)
	Ascorbic acid	198.42 ± 10.71 (6)**
Iron chelating activity	*Caesalpinia crista*	279.85 ± 4.72 (6)
	EDTA	1.27 ± 0.05 (6)***

^a^Unit of IC_50_ values of all activities is *μ*g/mL, except OH^•^ scavenging where unit is mg/mL. Data are expressed as mean ± SD. Data in parentheses indicate number of independent assays. EDTA represents ethylenediamine tetraacetic acid.

**P< .01; ***P < .001 versus *C. crista*.

**Table 2:** Effect of the methanolic extract of *C. crista* leaves on the activities of antioxidant enzymes and reduced glutathione content in liver of normal mice.

Tested parameter	Group I	Group II	Group III	Group IV
SOD (U/mg protein)	1.22 ± 0.54	2.61 ± 0.2**	2.96 ± 0.19***	3.37 ± 0.2***
CAT (U/mg protein)	0.9 ± 0.72	1.19 ± 0.09	1.5 ± 0.37*	1.66 ± 0.43*
GST (U/mg protein)	0.22 ± 0.05	0.31 ± 0.1	0.31 ± 0.08*	0.42 ± 0.08***
GSH (*μ*g/mg protein)	1.16 ± 0.04	1.43 ± 0.12*	1.78 ± 0.08***	2.1 ± 0.09***

Group I: control group; Group II: mice treated orally with the extract in a dose of 10 mg/kg body weight; Group III: mice treated orally with the extract in a dose of 50 mg/kg body weight; Group IV: mice treated orally with the extract in a dose of 100 mg/kg body weight. Results are expressed as mean ± SD for six mice in each group.

*P< .05, **P < .01, ***P < .001 compared with control.
